# A Novel Mutation in ACTG1 as the Probable Cause of Nonsyndromic Hearing Loss in Chinese Han Population

**DOI:** 10.1155/np/4050645

**Published:** 2026-08-27

**Authors:** Yajing Zhu, Tianyu Wang, Xianglan Sun, Bojian Lin, Huanhai Liu, Li Chen, Yin Cheng, Hu Peng

**Affiliations:** ^1^ Department of Otorhinolaryngology-Head and Neck Surgery, The Second Affiliated Hospital of Naval Medical University, Shanghai, China; ^2^ Department of Otolaryngology–Head and Neck Surgery, First Affiliated Hospital of Naval Medical University, Shanghai, China, smmu.edu.cn; ^3^ Department of Otolaryngology–Head and Neck Surgery, The Affiliated Xuzhou Municipal Hospital of Xuzhou Medical University, Xuzhou, Jiangsu Province, China

**Keywords:** ACTG1, Chinese Han population, nonsyndromic hearing loss

## Abstract

**Background:**

Hearing loss is the most common sensory nervous system defect in humans. Approximately half of hearing loss cases have a genetic etiology. At present, more than 300 genes and 1000 mutations have been identified that cause hereditary hearing loss (HHL). However, there are still a large number of unknown genes related to hearing loss.

**Objective:**

The purpose of this study was to analyze the clinical audiological characteristics of a family with hereditary nonsyndromic hearing loss (NSHL) and identify the mutation of deafness‐related genes.

**Material and Methods:**

A Chinese Han–dominant deafness family including six members was enrolled in our study. In addition to collecting detailed clinical data on the audiology of this family, we employed whole‐exome sequencing (WES) and Sanger sequencing to identify the key gene causing deafness.

**Results:**

The pedigree chart of the HHL family suggested that the disease might be a dominant genetic disease. The WES and Sanger sequencing indicated that a novel mutation (c.842C > G) occurred in the ACTG1 gene on the fifth exon, which caused the change of the coding protein (p.S281C). This variant cosegregated perfectly with the hearing loss phenotype: All affected members carried the heterozygous c.842C > G mutation, whereas the only unaffected family member showed wild‐type ACTG1. Multiple sequence alignment revealed that the p.Ser281 residue was highly conserved across vertebrate species. Bioinformatic analyses showed high conservation of p.Ser281 across species and predicted damaging effects on protein structure and function.

**Conclusions and Significance:**

On the basis of our research, we identified a novel pathogenic variant in the ACTG1 gene responsible for NSHL, which expands the mutational spectrum of ACTG1‐related hearing loss. Further in vitro and in vivo studies may be performed to elucidate the underlying mechanism by which ACTG1 contributes to hearing function, potentially providing intervention targets for gene therapy of HHL.

## 1. Introduction

Hearing loss was one of the most common sensory impairments in humans, affecting more than half a billion people worldwide [1, 2]. Deafness had extensive genetic heterogeneity. It was estimated that genetic factors accounted for ~50% of hearing loss and ~70% of hereditary hearing loss (HHL) was nonsyndromic hearing loss (NSHL) [3, 4]. About 20% of cases of NSHL were inherited in a dominant form, and the vast majority were caused by abnormalities in a single gene [2, 5]. In auditory development, cyto‐actin is required for stereocilia hair cell development [6]. γ‐Actin is specifically required for the maintenance and function of cytoskeletal structures in the ear [7]. It is involved in the mechanical transduction of sound waves to electrical signals, which are relayed to the brain in the form of hearing [8, 9].

The ACTG1 gene was located at 17q25.3 [10, 11], and ACTG1‐encoded γ‐actin is ubiquitously expressed in all cell types and serves as an essential component for maintaining cytoskeletal architecture and function. It also plays a critical role in the mechanotransduction process that converts sound waves into electrical signals [12, 13]. The γ‐actin fibers were highly conserved helical polymers. Previous studies have confirmed that multiple variants in the ACTG1 gene are closely associated with nonsyndromic sensorineural hearing loss. Pathogenic variants in ACTG1 can also cause Baraitser–Winter syndrome 2 (BRWS2; OMIM 614583), which may present with hearing loss accompanied by epilepsy, intellectual disability, distinctive facial features, and cardiac and renal involvement. Through whole‐exome sequencing (WES) and Sanger sequencing, we identified a novel variant (c.842C > G) in the ACTG1 gene, which was confirmed as the genetic etiology of NSHL in this family, thereby enriching the genotypic and phenotypic spectrum of ACTG1‐related hearing loss.

## 2. Materials and Methods

### 2.1. Subjects

A Chinese Han–dominant deafness family including six members was enrolled in this study. Five members of all took part in clinical evaluation and genetic testing in the Department of Otolaryngology‐Head and Neck Surgery, Second Affiliated Hospital of Naval Medical University. The evaluation included a detailed clinical interview, physical examinations, and clinical audiological examinations. Each participant signed a written informed consent. This study was approved by the ethics committee of Shanghai Changzheng Hospital. All experiments were performed in accordance with relevant guidelines and regulations.

### 2.2. Clinical Audiological Examinations

Auditory evaluations were performed including otoscopic examination, otoacoustic emission (OAE), and pure tone audiometry (PTA). The hearing thresholds of subjects were determined by the air‐conduction pure‐tone average thresholds ranging from 250 to 8000 Hz. The hearing level was classified as normal (<20 dB), mild (20–40 dB), moderate (41–70 dB), severe (71–90 dB), and profound (>90 dB), as we previously described [14]. Hearing thresholds reported in this study were averages of the right and left ears. Romberg testing and tandem gait were performed for vestibular function examination. Computerized tomography (CT) scan of the temporal bone was carried out for excluding inner‐ear anomalies. In addition, systematic examinations were performed to exclude BRWS2‐related syndromic features, including epilepsy, intellectual disability, distinctive facial dysmorphism, and cardiac or renal anomalies.

### 2.3. Genetic Analysis of Mutation Identification

Genomic DNA was extracted from whole blood of five family members using a blood DNA extraction kit (QIAamp DNA Blood Mini Kit, Qiagen, Shanghai). Firstly, the WES was performed on the DNA in five blood samples. After being tested by agarose gel electrophoresis, the exome chip (Agilent SureSelectXT Human All Exon V6 60 M) was used for sample database preparation and target region capture. After library qualified using Qubit and Agilent 2100 Bioanalyzer, sequencing was performed on Illumina HiSeq with 2 × 150 bp. The data quality was verified and compared with HG19 BWA standard sequence. Then, the candidate variation of exon region was detected by using GATK‐HaplotypeCaller. The screened highly pathogenic mutations were compared with the known deafness gene database and scored by Phenolyzer. Finally, the candidate mutation sites were selected for verification by Sanger sequencing. Variant filtering pipeline:

1. Variants with MAF > 0.01 in gnomAD, 1000 G, EXAC, and in‐house controls were excluded.
2. Only variants in deafness genes consistent with autosomal dominant inheritance were retained.
3. All other candidate variants were excluded; only ACTG1 c.842C > G remained. Sanger sequencing was performed on all family members to confirm the ACTG1 c.842C > G variant. The primers are as follows:

ACTG1‐F: ACCAACCCCTCGACTCACCTTGAT;
ACTG1‐R: TCCTTCCTGGGTAGGTGTTGTGAGCTA.

### 2.4. Bioinformatics Analysis

To evaluate the evolutionary conservation of the p.Ser281 residue, homologous protein sequences of ACTG1 were retrieved from representative vertebrate species, including humans, chimpanzees, mice, rats, cattle, dogs, chickens, zebrafish, and *Xenopus laevis*. Multiple sequence alignments were performed using ClustalX. The results demonstrated that the p.Ser281 residue was highly conserved across all analyzed vertebrates, suggesting its functionally important role in evolution.

Based on the human cytoplasmic γ‐actin structure (PDB ID: 5L2W) from the PDB database, three‐dimensional models of the wild‐type and c.842C > G mutant proteins were constructed using PyMOL 2.5, and the differences in hydrogen bonds and spatial conformation were compared between the two forms. In silico pathogenicity prediction was conducted using SIFT, PolyPhen‐2, MutationTaster, REVEL, and CADD. The detailed prediction results are shown in Table 1. As shown in Table 1, all five bioinformatic prediction tools consistently suggested that the ACTG1 c.842C > G (p.Ser281C) variant had damaging effects on protein structure and function, supporting its pathogenic potential. Finally, the variant was classified according to the ACMG/AMP–ClinGen criteria.

**Table 1:** Bioinformatic pathogenicity prediction results of the ACTG1 c.842C > G (p.Ser281C) variant.

Prediction tool	Score	Prediction result
SIFT	0.001	Deleterious
PolyPhen‐2	0.982	Probably damaging
MutationTaster	0.998	Disease causing
REVEL	0.912	Damaging
CADD	23.7	Pathogenic

## 3. Results

### 3.1. Clinical Feature

**Figure 1:**
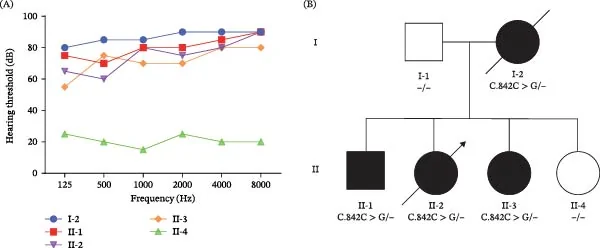
The clinical characteristics of the family with NSHL. (A) Audiogram of the proband (II‐2) and other family members (I‐2, II‐1, II‐3, and II‐4). (B) Pedigree chart of the Chinese Han family with ACTG1 mutation.

The proband was a 44‐year‐old female from Zhejiang Province, China. The present family members were investigated, and four of them were diagnosed with bilateral progressive profound sensorineural deafness by PTA (Figure 1A). No family members were affected by hearing loss prior to the four currently identified patients, and no obvious abnormalities in other organs or systems were identified in the patient. The characteristics of hearing loss in the family was bilateral profound deafness in the early stage, suggesting that the hearing loss may be genetically related. Besides, according to the hearing phenotypes, it is speculated that the HHL might be a dominant genetic disease caused by mutations from Ⅰ‐2 (Figure 1B). OAE was not detected in both ears of the four patients. Their temporal bone CT scan and cranial magnetic resonance imaging (MRI) showed no abnormalities. No vestibular dysfunction was complained by the patients. No obvious syndrome was observed in the physical examination. In addition, all patients had no established history of ototoxic drug use and no history of exposure to environmental noise.

### 3.2. Analysis of WES Data

**Figure 2:**
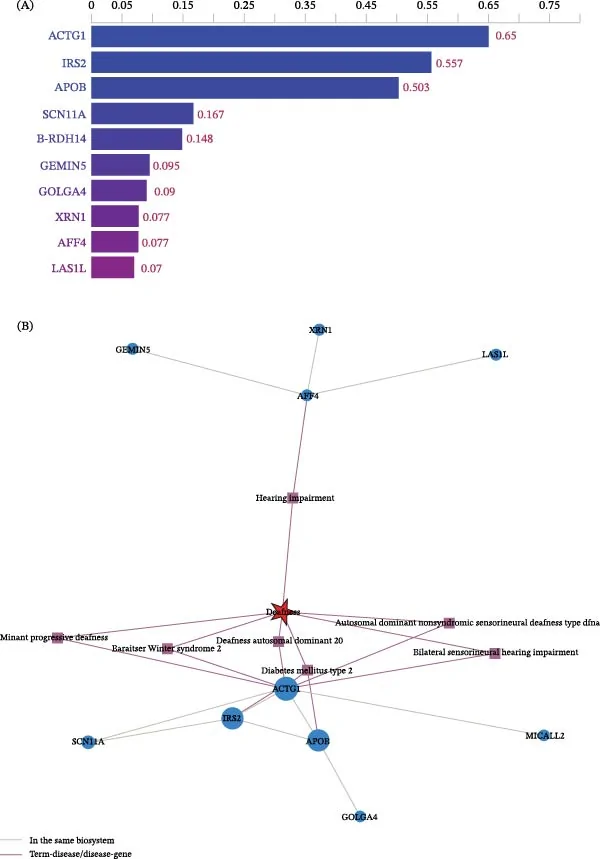
ACTG1 was recognized by WES. (A) Rank score of the potential pathogenic effect of the candidate mutation. The scores were predicted by Phenolyzer. (B) Gene‐disease projection networks. ACTG1 is closely associated with diseases that can cause deafness.

**Figure 3:**
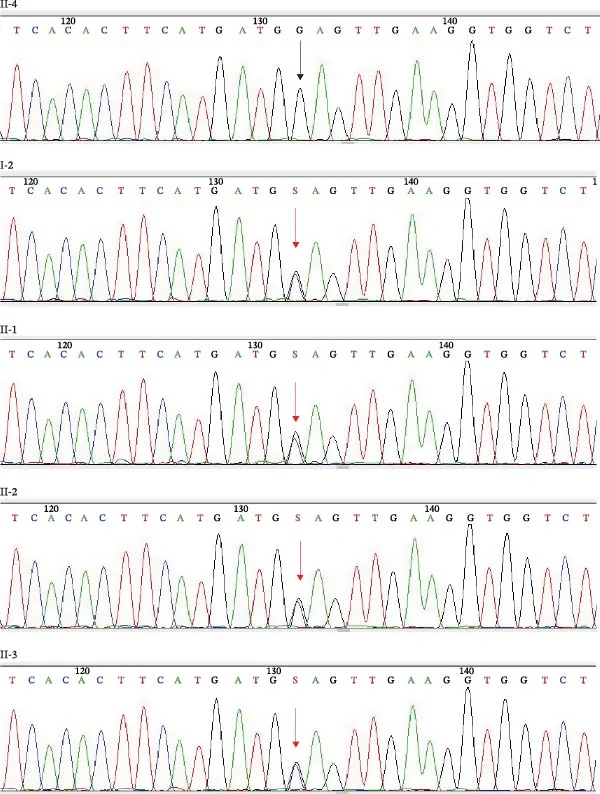
A novel mutation in ACTG1 was found by Sanger sequencing. The mutation (c.842C > G) in ACTG1 was identified in family members with NSHL, while the only member without NSHL did not carry the mutation.

**Figure 4:**
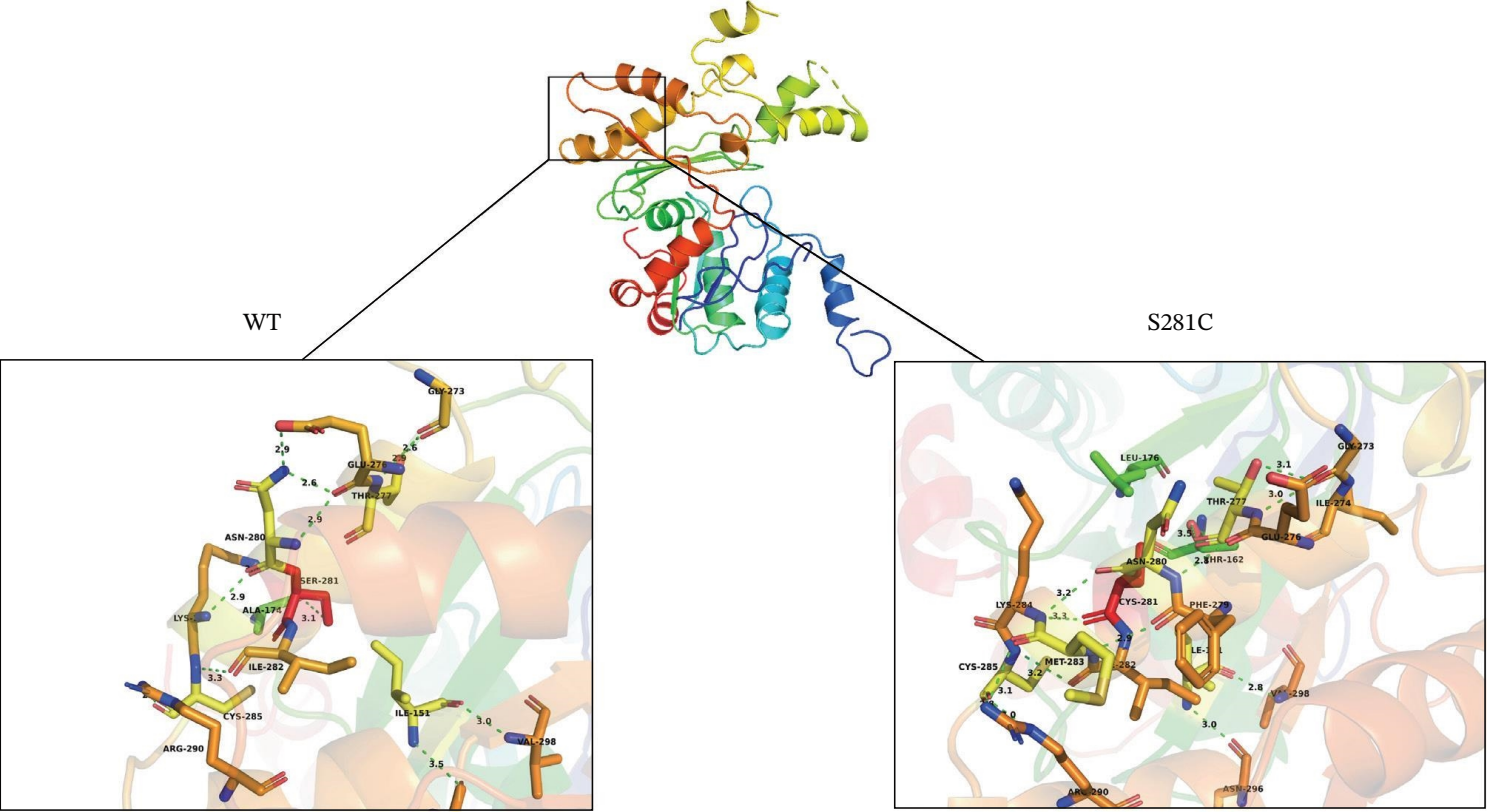
The changed protein structure of the ACTG1‐encoded γ‐actin. The mutation caused a change from serine to cysteine in the 281 site of coding protein.

By using WES in the five living members of the family, more than 466 mutant genes were found. In reference to the OMIM and HGMD database, the Phenolyzer (http://phenolyzer.wglab.org/) was utilized to conduct candidate gene sorting studies and analyze the scores obtained on the priority of genes associated with NSHL (Figure 2A). ACTG1 was the gene with the highest disease‐related priority score among the dominant mutated genes, suggesting that it might be highly associated with NSHL in the family. Additionally, ACTG1 was closely related to hearing capabilities according to the gene‐disease projection networks (Figure 2B). For each sample, ~12 Gb of data was obtained from WES with the Q30 being up to 93%. The data mapped to the targeted region had a mean depth of 117 fold, 97% of which was covered with at least 20×. Sanger sequencing confirmed that all four affected family members carried the heterozygous c.842C > G mutation in ACTG1. The only unaffected family member (Ⅱ‐1) showed homozygous wild‐type (C/C) at this locus and did not carry the mutation (Figure 3). Multiple sequence alignment demonstrated that the p.Ser281 residue in ACTG1 was highly conserved across diverse vertebrate species, including human, chimpanzee, mouse, rat, cattle, dog, chicken, zebrafish, and *X. laevis*. The high conservation indicated that this position is critically important for protein function. PyMOL was used to visualize the structural changes of the encoded protein caused by the mutation in ACTG1 (Figure 4). It is noteworthy that the variant was not seen in public databases dbSNP, EXAC, 1000 Genomes Project, and the in‐house databases of 200 Chinese Han normal–hearing controls.

## 4. Discussion

It was well known that causative missense mutations in the ACTB and ACTG1 genes could cause Baraitser–Winter cerebrofrontofacial syndrome (BWCFF) [6, 15]. The knockdown of ACTG1 suppressed the proliferation and migration of tumor cells such as skin cancer [16] and hepatocellular carcinoma [17]. In recent years, it was also reported that ACTG1 could participate in the pathological process of osteoarthritis (OA) [18] and intervertebral disc degeneration (IDD) [19]. Notably, hearing loss was still the most common disease related to ACTG1 [20]. Zhu et al. [11] and van Wijk et al. [10] clarified that the dysfunction of stereocilia induced by the mutation of γ‐actin, encoded by ACTG1, could lead to an autosomal dominant, progressive, sensorineural hearing impairment. It was agreed that mutations causing Mendelian diseases were mainly hidden in exons, and WES had become the most effective strategy to identify genetic diseases [21, 22]. Hitherto, various mutations in ACTG1 were revealed in consanguineous families from Europe, Italy, Taiwan, Czech Republic, Spain, Korea, Norway, America, Dutch Japan, and China [23–30]. However, a variation of c.842C > G (p.S281C) in ACTG1 was found to be a novel mutation associated with NSHL.

The ACTG1 gene encodes γ‐actin in auditory hair cells in the cochlea, which plays an essential role in maintaining the shape and function of the stereocilia [31]. In general, ACTG1‐related NSHL initially appeared at higher frequencies of hearing at the age of 14 and then aggravated to all frequencies [20]. However, the PTA of the family in our study showed a full‐frequency hearing loss, which may be attributed to the concealed clinical manifestation of high‐frequency hearing loss.

From the results of clinical features, WES, and Sanger sequencing, we recognized the new mutation of c.842C > G in ACTG1 to be the genetic cause of NSHL. The variant cosegregated with hearing loss and was absent in the unaffected family member, supporting its pathogenicity. The novel finding expanded our understanding of the phenotypes associated with ACTG1. Notably, our study might provide intervention targets for gene therapy of partial NSHL. On the basis of our research, further in vivo and in vitro experiments could be designed to analyze the internal mechanism of ACTG1 affecting the hearing.

Database annotations indicated that the variant corresponds to the dbSNP ID rs2143775658. This variant is extremely rare in the gnomAD population database and was not detected among the 200 normal controls enrolled in this study. ClinVar presents conflicting interpretations regarding the pathogenicity of this variant. The variant was identified as a de novo variant with confirmed paternal origin, while maternal validation could not be completed due to the mother’s death. The pathogenicity of the variant was evaluated according to the gene/disease‐specific ACMG/AMP interpretation criteria (specifications formulated by the ClinGen Variant Curation Expert Panel, VCEP), and this case meets the moderate evidence PM6. All five bioinformatic tools (SIFT, PolyPhen‐2, MutationTaster, REVEL, and CADD) predicted this variant to be damaging (Table 1). Combined with sequence conservation, protein structural alterations, and multiple bioinformatic prediction results, the variant was ultimately classified as likely pathogenic based on the ClinGen VCEP specifications with an evidence combination of PM6 + PM2 + PP3 + PP4.

## 5. Conclusion and Significance

On the basis of our research, we found a novel mutation in exon 5 of ACTG1 leads to the change of encoding protein p.S281C resulting in progressive profound deafness. This mutation was present in all affected members but absent in the only unaffected member of the family, confirming complete cosegregation with the disease phenotype. Hence, further in vivo and in vitro experiments could be designed to analyze the internal mechanism of ACTG1 affecting hearing, which may provide intervention targets for gene therapy of partial HHL.

## Funding

No funding was received for this study.

## Conflicts of Interest

The authors declare no conflicts of interest.

## Data Availability

The datasets generated and/or analyzed during the current study are available in the NCBI SRA repository, the Bioproject ID PRJNA951202 (http://www.ncbi.nlm.nih.gov/bioproject/951202).
